# Identification of Shared Genes Between Ischemic Stroke and Parkinson's Disease Using Genome-Wide Association Studies

**DOI:** 10.3389/fneur.2019.00297

**Published:** 2019-03-28

**Authors:** Wenjing Lang, Junjie Wang, Xiaofeng Ma, Nong Zhang, He Li, Pan Cui, Junwei Hao

**Affiliations:** ^1^Department of Neurology and Tianjin Neurological Institute, Tianjin Medical University General Hospital, Tianjin, China; ^2^Key Laboratory of Post-Neuroinjury Neuro-Repair and Regeneration in Central Nervous System, Tianjin Neurological Institute, Tianjin Medical University General Hospital, Ministry of Education and Tianjin City, Tianjin, China

**Keywords:** ischemic stroke, Parkinson's disease, genome-wide association studies, gene-based test, gene expression analyses

## Abstract

Ischemic stroke (IS) and Parkinson's disease (PD) are two neurological diseases that often strike individuals of advanced age. Although thought of as a disease of old age, PD can occur in younger patients. In many of these cases, genetic mutations underlie the disease. As with PD, stroke can also have a genetic component. Although many of the risk factors for IS are considered to be modifiable, a significant portion is not, suggesting that some of stroke risk factors may have a genetic origin. Large-scale genome-wide association studies (GWAS) have identified several IS and PD gene variants recently. Converging epidemiologic and pathological evidence suggests that IS and PD may be linked. However, it is still unclear whether these two conditions share a common mechanism. Here, we sought to determine the genetic mechanism underlying the possible association between IS and PD. We conducted a multi-step systemic analysis comprising (1) identification of IS and PD variants validated by known GWAS, (2) two separate gene-based tests using Versatile Gene-based Association Study 2 (VEGAS2) and PLINK, (3) a transcriptome-wide association study (TWAS), and (4) analyses of gene expression using an online tool in Gene Expression Omnibus. Our investigation revealed that IS and PD have in common five shared genes: *GPX7, LBH, ZCCHC10, DENND2A*, and *NUDT14*, which pass gene-based tests. Functionally, these genes are expressed differentially in IS and PD patients compared to neurologically healthy control subjects. This genetic overlap may provide clues on how IS and PD are linked mechanistically. This new genetic insight into these two diseases may be very valuable for narrowing the focus of future studies on the genetic basis of IS and PD and for developing novel therapies.

## Introduction

Stroke and Parkinson's disease (PD) are two neurological diseases that have great worldwide impact and that share certain clinical and pathological features. Stroke is a major cause of disability in many western countries. Moreover in 2015, it was the second leading cause of death, accounting for 11% of total deaths (6.3 million) (1, 2). Ischemic stroke (IS) accounts for up to 85% of all stroke cases. PD similarly affects many people and in 2015, it resulted in about 117,400 deaths globally (2, 3).

Both IS and PD have substantial genetic components. Evidence for a substantial genetic contribution for IS risk comes from genome-wide association studies (GWAS) and twin and family history studies (4–7). GWAS have uncovered risk loci for IS (4–7). Falcone et al. reviewed several common genetic variants of certain forms of IS that do not follow a clear Mendelian pattern of inheritance. These variants include *ABO, PITX2, ZFHX3, HDAC9, SUPT3H/CDC5L*, and *CDKN2A/CDKN2B* (6). Candidate-gene analyses and GWAS have identified a new locus at chromosome 6p25 (rs12204590, near *FOXF2*) that is related to all-stroke risk (8). Also, a new locus at chromosome 1p13.2 near *TSPAN2* was recently identified; this latter locus is related to large artery atherosclerosis (LAA)-related stroke (9). Many researchers suspect that several other variants are yet to be identified.

As with IS, emerging evidence shows that PD has a substantial genetic component (10–12). GWAS and linkage analysis have confirmed the role of genes involved in familial and sporadic forms of PD (13, 14). Analysis of five large-scale GWAS datasets from Europe and the USA has identified some risk SNPs (*p* < 5.00E-08) through meta-analysis of PD susceptibility genes, including *MAPT, SNCA, HLA-DRB5, BST1, GAK, LRRK2, SYT11, ACMSD, STK39, MCCC1/LAMP3*, and *CCDC62/HIP1R* (13). There is now abundant GWAS data on numerous phenotypes of various diseases. Simultaneous analyses of multiple phenotypes can increase the detection of shared pathways, a procedure that could prove to be fruitful for identifying common genes of IS and PD.

Converging molecular, cellular, genetic, and clinical evidence has been reported for IS and PD. In a large population-based study, Huang et al. (15) and Becker et al. (16) confirmed that PD is related to an increased risk of IS and vice versa, implying that the two diseases may share some pathological mechanisms or processes. One possible link between IS and PD may involve α-synuclein, especially oligomeric forms (17). Abnormal aggregations and form conversion of α-synuclein are thought to result from the induction of oxidative stress and may be the pathological basis for PD (18). α-synuclein appears to be similarly elevated in red blood cells of IS and PD patients, being significantly higher than that in healthy people (17). α-synuclein induces microglia-mediated neuroinflammation, and α-synuclein aggregation indirectly damages neurons (19–21). Taken together, it is reasonable to hypothesize that, although IS and PD are two very different diseases, they may share pathophysiological processes that link them at some level. Building on this hypothesized relationship, one might expect to detect common immune-related genetic risk factors.

While GWAS has been revolutionary in unraveling disease genetics in general, for IS and PD, a large proportion of genetic variants remain undiscovered, serving as a reminder that more work are necessary to identify other genes that contribute to the pathology of these two diseases. We hypothesize that combining analyses of genes identified from different gene-based tests may be a powerful approach for identifying genes shared by IS and PD. We tested this hypothesis by conducting two gene-based meta-analyses using VEGAS2 and PLINK on IS and PD data from GWAS. In addition, we examined the shared genes by TWAS, and further validated the shared gene data with gene expression analyses utilizing GEO datasets.

## Materials and Methods

### Participant Samples

#### PD GWAS Dataset

Pankratz et al. (22) originally analyzed the PD GWAS dataset. They also conducted a large meta-analysis on two new datasets obtained directly from the investigators who performed the original GWAS (23–25) and on publicly available GWAS data obtained from dbGaP (10, 26), PROGRNI/GenePD (23), NIA Phase I (26), NIA Phase II (10), HIHG (24), and NGRC (25). They designed a two-stage study, which comprised a discovery stage and an independent replication stage. All the datasets used in the discovery stage came from Caucasian PD patients who were diagnosed using standard UK Brain Bank criteria for PD (27). Since familial PD cases may have a stronger genetic contribution than sporadic PD cases (22), they additionally included data from cases with a family history of PD. Anyone with a PD onset age younger than 18 years was excluded from the study. They also removed data of cases that had a known pathogenic factor, such as two parkin mutations or single *LRRK2* mutations.

In the original publication, each study underwent rigorous quality assessment and data cleanup before performing imputation with MACH1.0 (28). To control population stratification, the researchers used principal component analysis. ProbABEL (https://cran.r-project.org/src/contrib/Archive/GenABEL/), a tool for genome-wide association analysis of imputed data, and METAL (http://www.sph.umich.edu/csg/abecasis/Metal), a tool for meta-analysis, were then used to analyze the data. Finally, we acquired summary PD GWAS statistics data from the discovery samples, which included 2,525,704 SNPs from 4,238 PD cases and 4,239 control cases [for additional details, see the original article (22)].

#### IS GWAS Dataset

The IS summary GWAS data was obtained from phase I of the METASTROKE collaboration, which consisted of 10,307 Caucasian IS cases and 19,326 Caucasian control cases (29). These cases came from 12 studies (ASGC, BRAINS, GASROS_affy, GASROS_illumina, GEOS, HPS, ISGS-SWISS, MILANO, VISP, WHI, WTCCC2-D, and WTCCC2-UK) with previously genotyped data. The etiologic stroke subtypes were classified according to the criteria of the Trial of ORG 10172 in Acute Stroke Treatment (TOAST) (30).

For all datasets, the researchers performed genotyping individually and quality controls using methods documented previously (30). Using 1000 Genomes phase I data, the researchers imputed the raw autosomal data following a genome-wide logistic regression analysis and a meta-analysis (31). In order to determine whether the effector alleles were identical, SNPs were analyzed across the cohort in a meta-analysis. Additionally, genomic control was also used for test statistics to correct for incidental inflation. Finally, we obtained summary IS GWAS data, including 9,541,572 SNPs [for additional details, see the original article (29)].

### Statistical Analysis

#### Gene-Based Testing Using VEGAS for GWAS Datasets

To test the IS and PD GWAS datasets, we calculated gene-based *p* values with VEGAS2 after assigning variants to genes. We chose a broad reference population group, having a European ancestry (1000G EURO). For gene boundary selection for SNP, we implemented option 5, “0kbldbin” (SNPs within this gene and SNPs in high LD outside of this gene with SNPs within this gene). In gene-based tests, distant SNPs with r^2^ > 0.8 with associated SNPs were usually not taken into consideration systematically, and yet ignoring LD might lead to deficiency of some valuable information, therefore we chose this larger gene boundary. For every gene definition, the gene-based test statistics were calculated by adding the *p* values of n SNPs after conversion to upper tail χ^2^ statistics with one degree of freedom (*df*). Under the null hypothesis, these should have a χ^2^ distribution with *n df*, if SNPs are in linkage equilibrium (LD) (32).

SNP correlation was modeled using ∑, a *n* × *n* matrix of LD (*r*) values estimated from a 1000 Genomes European reference population (32). We used this method because LD for the *n* SNPs scarcely occurs (32). Significance was calculated by comparing the sum of χ^2^ statistics for every gene with simulated repeats from a multivariate normal distribution, where the mean = 0 and variance = ∑ (32). The formula *p* = *r*+1/*m*+1 was used to calculate empirical *p* values for every gene, where *r* represents the number of instances in which the simulation statistic exceeds the observation data, and *m* represents the number of simulations (32). This gene-based test included all top SNPs (by default, all SNPs are considered). We submitted IS and PD variants to VEGAS2 separately, and then identified shared genes that were nominally associated in each disease separately (*P*_IS−GWAS_ < 0.05; *P*_PD−GWAS_ < 0.05) (33).

#### Gene-Based Testing Using PLINK for GWAS Datasets

To test IS and PD GWAS datasets, we used Fisher's method implemented in PLINK software (SET SCREEN TEST). If the performed tests are independent for each SNP, for a given gene, the combined Fisher's statistic

$$\begin{array}{l} x_{0}^{2}=-2\overset{N}{\underset{i=1}{\sum }}\text{ln }\left(p_{i}\right)\; \end{array}$$

follows a χ^2^ distribution, with 2*N df* under the null hypothesis. In this formula, *N* represents the number of markers (tests), and *p*_*i*_ (*i* = 1,…, *N*) represents the corresponding *p* values. If the tests are not independent, the statistic $x_{0}^{2}$ has mean *m* = 2*N* and variance (σ^2^) is

$$\begin{array}{l} \sigma^{2}=4N+\overset{N-1}{\underset{i=1}{\sum }}\overset{N}{\underset{j=I+1}{\sum }}cov\left(-2\text{ ln }\left(p_{i}\right),\;-2\text{ ln }\left(p_{j}\right)\right)\; \end{array}$$

In the formula above, *p*_*i*_ and *p*_*j*_ (*i, j* = 1, …, *N*) represent the *p* values for each test. The covariance (cov) can be calculated as

$$\begin{array}{l} \text{cov}\left(-2\text{ ln }\left(p_{i}\right),\;-2\text{ ln }\left(p_{j}\right)\right)=p_{ij}\left(3.25+0.75p_{ij}\right)\; \end{array}$$

where *p*_*ij*_ approximates the correlation between SNP_i_ and SNP_j_. These are the non-negative correlation coefficients between the two variables.

Thus, the significance of a complete set of non-independent tests is calculated as

$$\begin{array}{l} x^{2}=-2\overset{N}{\underset{i=1}{\sum }}\text{ ln }\left(p_{i}\right)\times \frac{4N}{\sigma^{2}}\; \end{array}$$

where *x*^2^ follows the central Chi-squared distribution, with 8*N*^2^ /σ^2^ as *df*.

This method was applied to the PD GWAS dataset and IS GWAS dataset using LD information from the HapMap CEU population. An approximate Fisher's test was used for all the SNPs in genes to combine *p* values (34). By combining a group of *p* values that were acquired from independent tests with the same null hypothesis, we found that the Fisher's method was asymptotically optimal to achieve overall significance (34). Genes with many of SNPs are well suited to using this approach (34–36). After performing calculations in PLINK, we identified shared genes that were associated with each disease (*P*_IS−GWAS_ < 0.05; *P*_PD−GWAS_ < 0.05).

#### Meta-Analysis of Shared Genes

We combined the two *p* values of the shared genes of IS and PD derived from VEGAS using the simplest meta-analysis method in GWAS: Fisher's method. We conducted the same meta-analysis for shared genes identified by PLINK. For a given gene, we chose the following formula for the statistic

$$\begin{array}{l} x^{2}=-2\overset{k}{\underset{i=1}{\sum }}\text{ ln }\left(Pi\right)\; \end{array}$$

where in the *ith* study, *P*_*i*_ is the gene's *p* value; and *k* represents the overall number of studies. Under *2k df*, *x*^2^ follows a χ^2^ distribution (37). We used the program R (https://www.r-project.org/) to finish the analysis.

#### Transcriptome-Wide Association Study

TWAS combines gene expression data with GWAS data to identify genes that could regulate the expression of complex traits in *cis*-action (38). We performed a validation of the shared genes using TWAS in different tissues to determine whether they played significant roles in expression-trait associations. The process of TWAS have been widely described in previous articles (38). Here, TWAS integrated pre-computed gene expression weights of whole blood and brain RNA-seq with GWAS data to estimate the associations of gene to traits. The reference data of whole blood comprised 1,264 samples of Cardiovascular Risk in Young Finns Study (YFS) in Finland (39), and the reference data of brain RNA-seq was collected from the dorsolateral prefrontal cortex of 452 samples from the CommonMind Consortium (CMC) (40).

#### Gene Expression Analyses

To bolster our gene-based testing results with biological functional data of shared genes and to further validate the shared genes of IS and PD, we used GEO2R (41), an online tool that can identify differentially expressed genes under different experimental conditions. This tool compares two or more sample groups in the Gene Expression Omnibus (GEO) database.

Gene expression data from analyses of peripheral whole blood of 39 IS patients and 24 non-stroke, neurologically healthy control subjects were obtained from GEO dataset GSE16561. Patients were recruited if they were ≥18 years and diagnosed definite IS by MRI. In addition, patients diagnosed hemorrhage and uncertain IS were excluded from the group. There were no significant differences in gender and race between patients and controls, but more vascular risk factors (such as hypertension, diabetes, etc.) were found in stroke subjects. More details about clinical characteristics were provided in the original report (42). The samples were analyzed on an Illumina HumanRef-8 Expression BeadChip. We also used another publically available stroke RNA expression dataset, GEO dataset GSE58294 (43). These data were derived from analyses of peripheral whole blood of 23 cardioembolic stroke patients and 23 vascular risk factor controls (VRFC). Race was not statistically significantly different between stroke cases and VRFC. More subject demographics were described in the original article (43).

For changes in gene expression in PD patients, global expression data derived from analyses of postmortem brain tissue were acquired from GEO dataset GSE20295 (44, 45). This dataset comprises three subseries (GSE20168, GSE20291, and GSE20292). The tissue blocks which were from three brain areas, prefrontal cortex area 9, the putamen, and the entire substantia nigra, were collected from 15 patients with neuropathologically confirmed PD and 15 controls without major brain disease. PD patients diagnosed with additional neuropathological disease were excluded from the study. Between PD and control groups, some variables, for example gender, which could greatly affect the expression profile of RNA in postmortem brain tissues, were matched closely and had no significant differences (*p* > 0.05). [for more details, see the original report (44, 45)]. The data were analyzed using an Affymetrix Human Genome U133A Array platform.

NCBI GEO can be used as a public repository for a variety of high-throughput experimental data. Currently, GEO contains nearly 140,000 samples and more than 3,000 different microarray platforms (46). GEO2R uses the GEO query (46) and Linear Models for Microarray Analysis (47) (limma) R packages from the Bioconductor project (https://www.bioconductor.org) to compare the processing data tables provided by the original submitter. Although our main goal was to identify the consensus genes (listed above) in each dataset, we also screened and ranked by significance all of the genes shared by IS or PD. The latter was done by assessing their differential expression relative to control subjects. The permutation allowed a null distribution of gene ranking per experiment. Then we evaluated whether the shared genes deviated significantly from the null distribution.

## Results

### Gene-Based Testing

#### Gene-Based Meta-Analysis With VEGAS2

Using VEGAS2, we assigned variants to genes and calculated *p* values for IS and PD datasets separately. From those analyses, we obtained 20,946 IS genes and 19,858 PD genes with at least two SNPs. We subsequently identified 75 genes that were nominally associated with each individual disease (*P*_IS−GWAS_ < 0.05; *P*_PD−GWAS_ < 0.05) and shared by IS and PD. Following meta-analysis of the 75 genes and Bonferroni correction with *p* < 3.33E-04 (*p* = 0.05/75/2), we ultimately identified nine shared genes between IS and PD: *GAK* (*p* = 1.24E-06 for IS and PD, 3.55E-02 for IS, and 2.00E-06 for PD); *MMRN1* (*p* = 1.82E-05 for IS and PD, 3.38E-02 for IS, and 3.70E-05 for PD)*; GPX7* (*p* = 2.70E-05 for IS and PD, 2.00E-03 for IS, and 9.52E-04 for PD); *NUDT14* (*p* = 3.65E-05 for IS and PD, 3.56E-04 for IS, and 7.40E-03 for PD); *LBH* (*p* = 4.55E-05 for IS and PD, 1.57E-03 for IS, and 2.13E-03 for PD); *ZCCHC10* (*p* = 5.68E-05 for IS and PD, 6.30E-03 for IS, and 6.75E-04 for PD)*; P2RX6* (*p* = 1.03E-04 for IS and PD, 6.23E-03 for IS, and 1.30E-03 for PD); *DENND2A* (*p* = 2.24E-04 for IS and PD, 2.42E-03 for IS, and 7.78E-03 for PD); and *LOC101928455* (*p* = 3.07E-04 for IS and PD, 2.69E-03 for IS, and 9.90E-03 for PD). Detailed information about these genes is provided in Table 1.

**Table 1:** Nine genes shared by ischemic stroke and Parkinson's Disease cases identified with VEGAS2 (adjusted *p* < 3.33E-04).

		**Ischemic stroke**		**Parkinson's disease**			
**Chr**	**Gene**	**nSNPs**	***p***	**nSNPs**	***p***	**χ^2^**	**Meta-analysis**
4	*GAK*	383	3.55E-02	79	2.00E-06	32.92	1.24E-06
4	*MMRN1*	142	3.38E-02	51	3.70E-05	27.18	1.82E-05
1	*GPX7*	19	2.00E-03	3	9.52E-04	26.34	2.70E-05
14	*NUDT14*	18	3.56E-04	10	7.40E-03	25.69	3.65E-05
2	*LBH*	102	1.57E-03	40	2.13E-03	25.22	4.55E-05
5	*ZCCHC10*	81	6.30E-03	21	6.75E-04	24.74	5.68E-05
22	*P2RX6*	51	6.23E-03	13	1.30E-03	23.45	1.03E-04
7	*DENND2A*	243	2.42E-03	53	7.78E-03	21.76	2.24E-04
2	*LOC101928455*	118	2.69E-03	50	9.90E-03	21.07	3.07E-04

*Chr, chromosome; nSNPs, number of single nucleotide polymorphisms*.

#### Gene-Based Meta-Analysis With PLINK

Using PLINK, we mapped IS and PD SNPs to genes and identified 16,724 IS genes and 16,610 PD genes with at least two SNPs. We subsequently identified 33 genes shared by IS and PD that were nominally associated with each individual disease (*P*_IS−GWAS_ < 0.05; *P*_PD−GWAS_ < 0.05). Following meta-analysis of the 33 genes and Bonferroni correction with *p* < 7.58E-04 (*p* = 0.05/33/2), we finally identified nine genes associated with the two diseases. Theses nine genes are *NUDT14* (*p* = 3.86E-05 for IS and PD, 3.58E-04 for IS, and 7.82E-03 for PD); *PARP3* (*p* = 5.28E-05 for IS and PD, 2.30E-03 for IS, and 1.71E-03 for PD); *GPX7* (*p* = 8.08E-05 for IS and PD, 2.50E-03 for IS, and 2.49E-03 for PD); *ZCCHC10* (*p* = 8.19E-05 for IS and PD, 7.22E-03 for IS, and 8.75E-04 for PD); *MEX3A* (*p* = 2.26E-04 for IS and PD, 4.54E-04 for IS, and 4.20E-02 for PD); *DENND2A* (*p* = 5.03E-04 for IS and PD, 5.51E-03 for IS, and 8.30E-03 for PD); *CRNN* (*p* = 6.31E-04 for IS and PD, 2.37E-02 for IS, and 2.48E-03 for PD); *RGS9BP* (*p* = 6.31E-04 for IS and PD, 3.74E-03 for IS, and 1.57E-02 for PD); and *LBH* (*p* = 7.44E-04 for IS and PD, 1.01E-02 for IS, and 7.01E-03 for PD). Detailed information about these genes is provided in Table 2.

**Table 2:** Nine genes identified with PLINK shared by cases with ischemic stroke and Parkinson's disease (adjusted *p* < 7.58E-04).

		***p***			
**Chr**	**Gene**	**Ischemic stroke**	**Parkinson's disease**	**χ^2^**	**Meta-analysis**
14	*NUDT14*	3.58E-04	7.82E-03	25.57	3.86E-05
3	*PARP3*	2.30E-03	1.71E-03	24.89	5.28E-05
1	*GPX7*	2.50E-03	2.49E-03	23.98	8.08E-05
5	*ZCCHC10*	7.22E-03	8.75E-04	23.94	8.19E-05
1	*MEX3A*	4.54E-04	4.20E-02	21.73	2.26E-04
7	*DENND2A*	5.51E-03	8.30E-03	19.99	5.03E-04
1	*CRNN*	2.37E-02	2.48E-03	19.48	6.31E-04
19	*RGS9BP*	3.74E-03	1.57E-02	19.48	6.31E-04
2	*LBH*	1.01E-02	7.01E-03	19.12	7.44E-04

*Chr, chromosome*.

#### Identifying Shared Genes Using Two Different Approaches

Due to differences in identifying shared genes with either the VEGAS2 or PLINK method, we expected that shared genes would be more strongly associated with the diseases if they met the gene-based testing criteria of both methods. By determining where the results of statistically significant genes obtained by the two methods intersected, we ultimately obtained five shared genes satisfying the gene-based testing conditions. These genes are *GPX7, NUDT14, LBH, ZCCHC10*, and *DENND2A*. Meanwhile 4 of these 5 genes were also shown to be significantly associated with the two diseases in different tissues through TWAS (*p* < 0.05). Detailed results were summarized in Table 3. We next sought validation through other functional analyses.

**Table 3:** Five genes identified with two methods were verified by TWAS.

		**Ischemic stroke**		**Parkinson's disease**	
**Chr**	**Gene**	***p*_**TWAS**_**	**Comparative tissue**	***p*_**TWAS**_**	**Comparative tissue**
1	*GPX7*	1.44E-02	Whole blood	1.10E-01	Whole blood
2	*LBH*	9.33E-02	Whole blood	3.16E-02	Whole blood
5	*ZCCHC10*	–	–	–	–
7	*DENND2A*	8.58E-04	Brain RNA-seq	8.55E-03	Brain RNA-seq
14	*NUDT14*	4.31E-04	Brain RNA-seq	1.85E-02	Brain RNA-seq

*Chr, Chromosome; –, Not available*.

#### Gene Expression Analyses of Identified Genes

We further investigated whether these five shared genes were differentially expressed in IS and PD patients compared to neurologically healthy control subjects. We applied the Bonferroni-corrected statistical test at a significance of *p* < 0.01 (*p* = 0.05/5) and log_2_-fold change (logFC) to measure changes in the levels of gene expression. Compared to control subjects, in IS patients we detected significantly altered transcript levels of *GPX7* (*p* = 1.46E-07); *NUDT14* (*p* = 9.00E-03); *LBH* (*p* = 5.45E-06) in the GEO dataset GSE16561; and *ZCCHC10* (*p* = 3.87E-08) in the GEO dataset GSE58294 (Table 3). In PD brains, we found significantly altered expression levels of *GPX7* (*p* = 8.81E-03); *LBH* (*p* = 7.05E-03); *ZCCHC10* (*p* = 7.01E-03); and *DENND2A* (*p* = 9.77E-03) in GEO dataset GSE20295 (Table 4). It is remarkable that transcript expression of *GPX7* (GSE16561: logFC = −0.499; GSE20295: logFC = −0.802) and *LBH* (GSE16561: logFC = −0.553; GSE20295: logFC = −1.058) was significantly decreased in both IS patients and PD patients compared to the corresponding control groups.

**Table 4:** Shared genes in patients with ischemic stroke or Parkinson's disease vs. their corresponding healthy controls.

	**Ischemic stroke vs. control**				**Parkinson's disease vs. control**	
	**GSE16561**		**GSE58294**		**GSE20295**	
**Gene**	***p***	**logFC**	***p***	**logFC**	***p***	**logFC**
*GPX7*	1.46E-07	−0.50	3.59E-01	−0.10	8.81E-03	−0.80
*NUDT14*	9.00E-03	−0.22	2.53E-02	0.17	–	–
*LBH*	5.45E-06	−0.55	7.92E-01	0.03	7.05E-03	−1.06
*ZCCHC10*	2.92E-01	0.13	3.87E-08	0.85	7.01E-03	−0.84
*DENND2A*	4.18E-01	0.08	2.27E-02	0.19	9.77E-03	−0.95

–*, Not available*.

## Discussion

It has been widely reported that IS and PD share various pathological and clinical features (17, 48, 49). However, until now, the underlying genetic relationship between the two diseases has remained unclear. Previous studies have investigated IS and PD susceptibility genes independently and separately through analysis of IS and PD GWAS datasets and by performing independent linkage analyses (6, 8, 9, 13). Despite those efforts, a large proportion of genetic variants related to IS and PD remain undiscovered. What steps can be taken to identify these variants? We hypothesized that combining the findings from both IS and PD GWAS would lead to the identification of new shared variants. In the current study, we performed two gene-based tests on IS and PD GWAS datasets, examined the shared genes by TWAS and then conducted gene-expression analyses for validation. Through our analysis, it is significant to identify that IS and PD have shared genes, which explain shared pathogenesis between them to some extent. What's more, it is also consistent with a previous large study that, not only did PD patients have more frequent history of stroke than matched groups without PD, but the incidence rates of IS were also increased for PD patients compared with PD-free groups (16).

Through the two gene-based meta-analyses, we identified five new genes shared by IS and PD, which were further examined by TWAS. These five genes are *GPX7, NUDT14, LBH, ZCCHC10*, and *DENND2A*. Although we have not replicated published findings on IS/PD risk genes, these five shared genes we identified here have been studied extensively, and many experiments show that they play important roles in the pathogenesis of IS and PD (50, 51). GEO2R analyses also confirmed that expression level of these five genes in IS patients was different to that in control subjects. The same was true regarding the expression of these genes in PD patients and control subjects. Taken together, these results suggest that various related genes may underlie certain aspects of the pathogenesis of IS and PD.

### Glutathione Peroxidase 7 *(GPX7)*

*GPX7*, also known as *NPGPx*, is a member of the glutathione peroxidase (GPX) family of enzymes, which function to reduce oxidative damage (52). Ectopic expression of *GPX7* inhibits H_2_O_2_-induced toxic effects, which is consistent with its essential role in reducing oxidative stress (52). *GPX7* also transmits endoplasmic reticulum (ER) oxidative stress signals through the formation of disulfide bonds. This signal activates downstream ER glucose-regulated protein 78 (GRP78) and enhances its chaperone activity. Consistently, *GPX7*-knockout mice have been shown to accumulate reactive oxygen species, and they have a significantly shortened lifespan (53). Thus, it is clear that *GPX7* plays an important role as a stress sensor, functionally contributing to the attenuation of ER oxidative stress damage.

Mounting evidence confirms that ER stress contributes to the pathogenesis of PD and IS (51, 54, 55). Coppola-Segovia and collaborators have shown that model mice constructed to develop ER stress exhibit by injection of tunicamycin were induced PD features, such as dopamine neuronal death, increased astroglial reactivity, and extensive oligomerization of α**-synuclein (55). These features reinforce the notion that ER stress could play a pivotal role in the pathogenesis of PD (55). ER stress also appears to contribute to the pathogenesis of IS. During stroke, the unfolded protein response (UPR) signaling pathway is initiated by protein misfolding in energy-starved neurons, which is associated with the toxic effects of reperfusion (54). *GRP78* mainly regulates the UPR signaling pathway. The UPR signaling pathway plays an important role in attenuating ER stress by lessening protein translation, increasing folding capacity, and promoting ER-associated degradation and expansion of the ER membrane (56, 57). *GPX7* has also been shown to enhance the chaperone activity of *GRP78* to attenuate ER stress in transgenic animals (53).

In summary, the involvement of ER stress in pathogenesis of both IS and PD has been confirmed in many studies. *GPX7* could affect ER stress by direct regulation or by changing GRP78 indirectly, and hence participate in the pathological process of both diseases. We predict that it might be helpful in therapies of both diseases by interfering with *GPX7* to change ER stress. We need more validation studies in future.

### Limb-Bud and Heart *(LBH)*

*LBH* is a highly conserved, tissue-specific transcriptional regulator that plays a key role in the embryonic development of vertebrates (58–60). In epithelial development and cancer, *LBH* is a direct target gene for the canonical Wingless/Int (Wnt) signaling pathway (61). The relationship between the Wnt pathway and PD or IS has been widely reported (50, 62).

There is sufficient number of studies now suggesting that the Wnt signaling pathway is critical for the normal functioning of midbrain dopaminergic neurons. A growing number of genes that encode components of the Wnt pathway are involved in the development of dopaminergic neurons in the midbrain (63, 64), a region of early neuronal degeneration in PD, which is a pathological hallmark of the disease (65). In recent years, studies have revealed that the pathogenesis of PD can be traced back to gene mutations. Surprisingly, the Wnt signaling pathway has links with a striking number of PD susceptibility genes, such as *LRRK2* (66), *PARK2* (67), *VPS35* (68), *Nurr1* (69), *GSK3*β (70), and *WNT3* (71). *LRRK2*, for example, has been suggested to play a central role in the canonical Wnt pathway, and mutations in *LRRK2* decreases pathway activity (66). Taken together, it is reasonable to think that deregulation of the Wnt pathway might be an important precursor to the pathogenesis of PD. Does deregulation of the Wnt pathway make IS more likely?

Pathologically, stroke is the culmination of various insults to the vasculature. Recent work has shown that the Wnt pathway is involved in the development of central nervous system blood vessels, formation of the blood-brain barrier, and protection of injured endothelial cells (72, 73). In addition, GWAS analysis of IS patients and controls show that gender is implicated in the etiology of stroke (74), and male-specific stroke genes have been shown to be associated with the Wnt pathway (74). Furthermore, the Wnt pathway is involved in neuroinflammation, and it is important for neurogenesis (72, 75); these two processes are involved in PD and stroke. As *LBH* has been shown to be associated with other inflammatory disorders such as autoimmune diseases, in particular, rheumatoid arthritis (76), it might play a valuable role in IS and PD.

In general, the Wnt pathway has been demonstrated involving in both IS and PD susceptibility pathways by GWAS analysis. Numerous experiments have likewise demonstrated that Wnt signaling pathway is involved in the development of central nervous system, which comprises an important part of the pathological process of IS and PD. While *LBH* acts as a direct target gene for Wnt pathway, it is possible that LBH plays a role in the common pathogenesis of PD and IS.

### Zinc Finger CCHC Domain Containing 10 *(ZCCHC10)*

*ZCCHC10* was found to be closely associated with IS and PD in our analyses. However, to date, the function of *ZCCHC10* has revealed few links to IS, PD, or any other human diseases. One study suggested that *ZCCHC10* interacts with tumor protein p53 *(TP53)*, LUC7 like 2 (*LUC7L2*), peptidylprolyl cis/trans isomerase *(PIN1)*, and eukaryotic translation elongation factor 1 alpha 1 *(EEF1A1)* (77). One of *ZCCHC10*'s interaction partners, *EEF1A1*, plays an important role in the ability of monocyte locomotion inhibitory factor (MLIF) to protect the brain from ischemic damage. Knockout of *EEF1A1* attenuates MLIF's inhibitory effects on the expression of inflammatory molecules and ultimately reduces the protective effect of MLIF on IS (78). *EEF1A1* was identified to be an attractive candidate gene for PD as well (79). Furthermore, another *ZCCHC10*-interacting partner, *PIN1*, has been found to be dramatically upregulated in the substantia nigra of PD patients and to have a proapoptotic role in the pathophysiological mechanisms of PD (80). Thus, *ZCCHC10* could be an attractive gene by interacting with *EEF1A1/PIN1* for unraveling the pathophysiological relationship between IS and PD to some extent.

### DENN Domain Containing 2A *(DENND2A*)

*DENND2A* is a member of the *DEEND2* gene family and has been shown to be a specific guanine nucleotide exchange factor (GEF) for Rab9, which is involved in trafficking between the trans-Golgi network (TGN) and late endosomes (81). Although the function of *DENND2A* has rarely been reported to be relevant to diseases, Rab9-dependent mitophagy has been shown to contribute to heart disease (82). Recently mitophagy has been reported to play important roles in IS and PD (82–84). *DENND2A*, therefore, might be a promising gene by playing a part in mitophagy for determining the etiology of PD and IS.

### Nudix Hydrolase 14 *(NUDT14)*

*NUDT14* is one member of the 24 Nudix hydrolase genes of the human genome (85). *NUDT14* is proposed to be involved in the control of glycogen metabolism, where it modulates UDP-glucose levels during glycolipid and glycoprotein synthesis (86). A recent study showed that *NUDT14* could affect viral DNA replication by interacting with human cytomegalovirus RL13 (87). However, the function of *NUDT14* has few connections with IS or PD. In our study, *NUDT14* was identified as a candidate gene related to the pathogenesis of PD and IS. Additional research is needed in order to confirm this.

### Choice of Correction Method

As for the gene-based analysis, we used a more liberal cutoff genetic association *p* < 0.05 as the criterion for determining genes associated with IS or PD, rather than the multiple comparisons according to some considerations. For some complex disorders, the effect size of individual genetic variants is usually modest, which suggests that individual genetic variants could account for a minimal fraction of heritability of complex traits and genetic risk (88). Association signals for complex traits tend to be propagated throughout most of the genome, comprising genes which are not significantly connected to disease (89). In order to capture disease-related genes more comprehensively, we chose genes with nominal associations (*p* < 0.05) (33, 90). In addition, we obtained 20,946 IS genes and 19,858 PD genes with corresponding *p* values through VEGAS2. If we select Bonferroni correction for multiple test comparisons, the adjusted *p* value of IS genes should be < 0.05/20946 = 2.39E-06 and only 1 gene passes the correction, meanwhile, the adjusted *p* value of PD genes should be < 0.05/19858 = 2.52E-06 and 11 genes are with this significance level. If we take the same correction method for genes and *p* values obtained from PLINK software, 9 PD genes and 1 IS gene are with the significance level (*P*_*IS*_ < 2.99E-06, *P*_*PD*_ < 3.01E-06). Thus, we chose genes that were nominally associated in each disease for following analysis. For the obtained share genes, we performed Bonferroni correction for multiple test comparisons and genes with the significance level were verified as associated with both diseases.

### Limitations

The present study has some limitations despite of these interesting results. Since the original datasets were derived from patients who received clinical diagnosis of IS and PD, misdiagnosis could have potentially influenced on our results. In addition, we could not access the original SNP genotype data, we had to use summary data from IS and PD GWAS, which prevented us from using a polygenic risk score or BLUP method to address the shared genetics of complex traits and could have affected our results. We will improve our future work, when the original data is available to us. Besides, we utilized a small size of PD GWAS sample, compared with IS sample, which raised the possibility that the findings might be driven primarily by the IS sample. Moreover, as the multiple testing corrections we used in our statistical analyses may be insufficient to explain all biases, permutation testing should be used to adjust the results at the single SNP level. What's more, the data from different tissues for PD and IS might be a potential limitation to the results. We will further expand the size and tissues of expression data in the future. Furthermore, we lacked transcriptomic and epigenetic data, which may contribute to the identification of more potential causal mechanisms and associations. Finally, we did not further analyze the relationship between IS subtypes and PD. There are still some differences among IS subtypes, even though the pathological processes underlying each subtype have a certain degree of commonality.

## Conclusions

In conclusion, for two GWAS datasets for IS and PD, we used two gene-based testing methods and gene-expression analyses to identify several genes that are associated with neuroinflammation and neuro-immunity and that are expressed differentially in IS patients and PD patients. Based on previous work (91, 92), our outcomes support the hypothesis that IS and PD may be linked through shared neuroinflammation- and neuroimmune-related genes.

## Author Contributions

WL and JH conceived and designed the research for PD and IS; WL analyzed data, and wrote the manuscript; JW revised the manuscript; NZ, HL, and PC collected data and provided technical guidance; XM conceptualized the analysis and supervised the research. The final version was approved for submission by all listed authors.

### Conflict of Interest Statement

The authors declare that the research was conducted in the absence of any commercial or financial relationships that could be construed as a potential conflict of interest.
